# The Nurr1 ligand indole acetic acid hydrazide loaded onto ZnFe2O4 nanoparticles suppresses proinflammatory gene expressions in SimA9 microglial cells

**DOI:** 10.1038/s41598-024-64820-z

**Published:** 2024-06-17

**Authors:** Raneen Qasim, Tuqa Abu Thiab, Tareq Alhindi, Afnan Al-Hunaiti, Amer Imraish

**Affiliations:** 1 Department of Biological Sciences, School of Science, The University of Jordan, Queen Rania Al-Abdullah Street, Amman, 11942 Jordan; 2 Department of Chemistry, School of Science, The University of Jordan, Queen Rania Al-Abdullah Street, Amman, 11942 Jordan

**Keywords:** 3-Indole acetic acid hydrazide, Nanoparticles, Nurr1, Anti-inflammatory, Microglia, Cell biology, Immunology, Molecular biology, Neuroscience

## Abstract

The nuclear receptor-related factor 1 (Nurr1), an orphan nuclear receptor in microglia, has been recognized as a major player in attenuating the transcription of the pro-inflammatory genes to maintain CNS homeostasis. In this study, we investigate Nurr1 trans-repression activity by targeting this receptor with one of the indole derivatives 3-Indole acetic acid hydrazide (IAAH) loaded onto zinc iron oxide (ZnFe_2_O_4_) NPs coated with PEG. XRD, SEM, FTIR, UV–Vis spectroscopy, and DLS were used to characterize the synthesized IAAH-NPs. The anti-inflammatory properties of IAAH-NPs on LPS-stimulated SimA9 microglia were assayed by measuring pro-inflammatory cytokine gene expressions and protein levels using RT-PCR and ELISA, respectively. As a result, IAAH-NPs showed an ability to suppress pro-inflammatory genes, including IL-6, IL-1β, and TNF-α in LPS-stimulated SimA9 via targeting Nurr1. The current study suggests that ZnFe_2_O_4_ NPs as a delivery system can increase the efficiency of cellular uptake and enhance the IAAH ability to inhibit the pro-inflammatory cytokines. Collectively, we demonstrate that IAAH-NPs is a potential modulator of Nurr1 that combines nanotechnology as a delivery system to suppress neuroinflammation in CNS which opens a window for possible ambitious neuroprotective therapeutic approaches to neuro disorders.

## Introduction

Neuroinflammation is orchestrated by a group of glial cells that are coordinated in the response mechanism mediated by the hallmark mediators of neuroinflammation such as tumor necrosis factor (TNF)-α, interleukin (IL)-1β, and IL-6^1^. Typically, glial cells include oligodendrocytes, astrocytes, and microglia. However, the main player in neuroinflammation is microglia that first face the stimuli and then start the production of pro-inflammatory cytokines^2^. Activation of microglia initiates an immune response by secretion of several pro-inflammatory cytokines such as TNF-α, IL-1β, and IL-6 that have been detected in several neurodegenerative diseases which highlight the essential role of microglia in neuroinflammation^3^.

Retrieving central nervous system (CNS) homeostasis requires adequate inflammation that facilitates the healing process; however, excessive inflammation is associated with neurological disorders. Microglia-mediated inflammation is a fundamental aspect that regulates the immune response in CNS where these cells can suppress neuroinflammation by interrupting signaling pathways like nuclear factor (NF)-κB or targeting specific proteins such as Nurr1^2^. Nuclear receptor subfamily 4 group A member 2 (NR4A2), also known as Nurr1 has been witnessed huge attention by researchers due to its role in neuroinflammation suppression^4^. This receptor belongs to the NR4A nuclear receptor subfamily that also includes another two members (Nur77) and (NOR1) where these nuclear receptors are classified as early response genes that respond to multiple signals and interestingly can sense and respond rapidly to alterations in the cellular environment^5^. Nurr1 as a transcription factor is identified as an orphan receptor with an unknown ligand; therefore, several attempts have been made to target this receptor to inhibit the transcription of pro-inflammatory cytokine genes. For instance, amodiaquine (AQ) and chloroquine (CQ) were examined for their anti-inflammatory effects in lipopolysaccharide (LPS)-stimulated BV2 microglia that showed a reduction of TNF-α, IL-6, and IL-1β, but lack of specificity to this receptor was a hurdle besides low potency^6^. In addition, C-DIM12 [1,1-bis(3-indolyl)-1-(p-chlorophenyl) methane], an Indole derivative, exhibited an anti-inflammatory effect by targeting Nurr1 in microglia but it lacked selectivity^7^. In the meantime, the notion of using other indole derivatives loaded onto delivery materials and targeting Nurr1 has not been proposed yet. Collectively, Nurr1 is deemed to be a transcription factor that plays critical roles in neuron maintenance, survival, differentiation, and enhancement of anti-inflammation in non-neuronal cells; therefore, targeting this significant receptor will open the window for promising pharmacological neurotherapeutic approaches to maintain homeostasis in CNS.

Recently, nanoparticle (NP) technology has become prevalent and widely used in different aspects^8^. Regarding medical evolution, NPs count as a delivery system for different treatments because of their small size, easy manipulation, and ability to target specific tissue; the classical size of NPs is 1-100 nm with an upper limit of approximately 1000 nm^9,10^. Few approaches were made to target microglia in attempts to reduce neuroinflammation and find treatments for neurodegenerative disorders; however, their toxicity was a concern in the long term^11^. Iron oxide nanoparticles (IONPs) were found to be significantly engulfed by microglia confirmed by confocal imaging. Mechanistic studies revealed that exposure to IONPs attenuated the production of IL-1β mediated by inhibiting the secretory lysosomal pathway of cytokine processing, but not TNF-α in LPS-stimulated microglia. In addition, no cytotoxicity was observed after exposure to IONPs in primary microglial cells^12^. In another study, zinc iron oxide (ZnFe2O4) NPs showed an anti-inflammatory effect in LPS-stimulated RAW 264.7 macrophages with moderate toxicity which gives an insight into a further treatment regarding inflammation^13^.

In this study, we synthesized one of the indole derivatives IAAH loaded into ZnFe_2_O_4_ NPs and coated with PEG. ZnFe2O4 NPs and loaded IAAH were characterized using X-ray diffraction (XRD), Scan electron microscope (SEM), UV–visible spectroscopy, and dynamic light scattering (DLS). IAAH-NPs are believed to exhibit an anti-inflammatory effect on LPS-stimulated SimA9 microglia by activating Nurr1 receptor.

## Materials and methods

### Molecular docking simulations

The protein data bank (PDB) file (6DDA) of Nurr1 protein was downloaded from the PDB site. A single peptide chain was used for docking simulations using Autodock 4.2.6^14^. Water molecules were deleted, and polar hydrogen atoms were added, then Kollman charges were calculated, and atoms were assigned AD4 type using mgltools_1.5.6^15^. Ligands MOL2 files were constructed using their chemical formula via the (molview.org/) server, then converted to PDB files using BIOVIA Discovery Studio Visualizer. Blind docking simulations were carried out with rigid protein and flexible ligands, where the grid box encompassed the whole protein structure allowing us to explore possible binding sites. From these docking simulations, we obtained several models of the ligand binding site and its binding energy. The binding site of each ligand was visualized by PyMol software, the models where the ligands docked to the reported ligand binding sites reported in the literature were selected, and the final figures were prepared using BIOVIA Discovery Studio Visualizer^14,16^.

### ***Preparation and characterization of ZnFe***_***2***_***O***_***4***_***-NPs***

A 20 ml aqueous solution of 1 mM iron (III) chloride and 1 mM Zinc Nitrate was prepared using nano water. Then, 2 M oxalic acid was slowly added to the aqueous iron solution using the titration method. The reaction was stirred for 1 h at room temperature. Afterwards, the pH of the reaction was monitored and adjusted to be between 10 and 12 using NaOH solution. The product was then centrifuged, oven-dried at 80 °C for 4 h and washed with 20 mlx3 distilled water to remove any excess reagents such as NaOH or oxalic acid residues. The solid material was calcinated at 900 °C. Infrared spectra were recorded on KBr pellets in the 4000–400 cm^−1^ ranges on a Nicolet Avatar 360 FTIR spectrometer. The crystallographic structure of ZnFe_2_O_4_ nano-ferrites was determined from the Powder X-ray diffraction (P-XRD, mnochromatic Cu-Kα radiation, nickel filter, 40 kV, 30 mA using Shimadzu XRD-7000, Japan) pattern, while the particle morphology and size distribution was determined with the aid of electron microscopy imaging (SEM, JOEL 6400 and FEI QUANTA 200, Japan).

### Capping of IAAH

**Figure 1:**
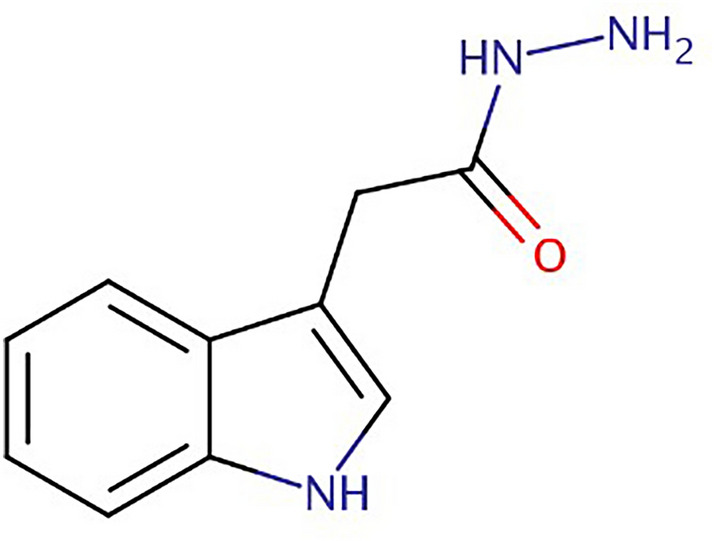
The structure of 3-Indole acetic acid hydrazide (IAAH).

3-Indole acetic acid hydrazide (IAAH), shown in Fig. 1, was treated with 200 ml of distilled water containing 0.5 g of ZnFe_2_O_4_ and mixed with 100 ml of 2% wt of acetic acid/PEG solution (100 ml/1.5 g), respectively at room temperature. The PEG cross-linker was stirred vigorously for 1 h at 45 °C and later centrifuged to obtain PEGylated IAAH-ZnFe_2_O_4_. The SEM micrographs of a fractured surface of the samples were recorded using JEOL 6400 and FEI QUANTA 200. To measure the particle size, polydispersity index (PDI), and zeta potential, a DLS Malvern Zetasizer (Malvern Instrument) was utilized, and size measurements were performed at room temperature. The capping efficiency was measured using UV–visible spectroscopy.

### Cell culture and maintenance

A spontaneously immortalized microglia (Sim-A9) cell line, isolated from a primary glial culture of postnatal murine cerebral cortices, was purchased from (ATCC® CRL-3265™, Manassas). The cells were maintained in Dulbecco’s Modified Eagle Medium/F12 (DMEM/F12, PAN BIOTECH) supplemented with 1% L-glutamine, 1% HEPES buffer, 10% heat-inactivated fetal bovine serum; (FBS, PAN BIOTECH), 5% horse serum; (HS, Gibco), and 1% penicillin–streptomycin (Gibco) in a 5% CO_2_ humidified incubator at 37 °C.

### Cytotoxicity assay

Sim-A9 cells were seeded in 96-well plates at a density of 9 × 10^3^ cells/well in complete growth media and incubated at 37°C for attachment overnight. The next day, the cells were treated with serial dilutions of IAAH-NPs, 100, 50, 25, 12.5, 6.25, and 3.125μg/ml. Alongside, control cells were treated with media alone. Treatment lasted for 72h, and half-maximal inhibitory concentration (IC_50_) was measured using the colorimetric MTT assay (Cell Titer 96 ® Non-Radioactive Cell proliferation assay, Promega). Plates were read at 570nm, and IC_50_ values were statistically calculated using GraphPad Prism 8 software.

### Cell treatment

To examine the anti-inflammatory effect of IAAH-NPs, SimA9 cells were seeded at 10 × 10^4^ cells/well in 12-well plates and allowed to attach overnight. The next day, three selected concentrations of IAAH- NPs were used for treatment 20, 10, and 5µg/ml which are 1/2.5, 1/5, and 1/10 values of IC_50_ (50.13 ± 10.53 µg/ml) respectively for 1h in serum-free media (SFM) before being treated with 100 ng/mL of LPS for 6h. Naked IAAH was tested to compare the ability of the nanoparticles to increase the efficiency of IAAH and dexamethasone was used as a positive anti-inflammatory control with a concentration of 40µg/ml.

### RNA extraction and gene expression analysis

Total RNA was isolated from SimA9 cells using a TRIzol reagent (Invitrogen). Total RNA concentrations were then measured using a Qubit 4 Fluorometer (Invitrogen) at 260 nm. The total RNA extraction was converted to cDNAs via Prime Script ™RT Master Mix Kit (Takara) following the manufacturer’s instructions. The relative expression levels of each gene were measured using a SYBR Green master mix in Quant Studio 1 cycler (Applied Biosystems). Specific primers with sequences are described in Table 1**.** Next, ΔΔCT was calculated using Excel, and results were analyzed using GraphPad Prism 8 software. The GAPDH gene was used as a reference gene in the experiment.

**Table 1:** Primers used for RT-PCR.

Gene	Primers sequences
TNF-α	F: 5′ GGCCTCCCTCTCATCAGTTC3′
	R: 5′GGTGGTTTGCTACGACGTG3′
IL-1β	F: 5′ TGCCACCTTTTGACAGTGATG3′
	R: 5′CCCAGGTCAAAGGTTTGGAGG3′
IL-6	F: 5′ TCTCTGCAAGAGACTTCCATCC3′
	R: 5′TGAAGTCTCCTCTCCGGACTT3′
Nurr1	F: 5′ GACCCGGGCTCCTCTGCTC′
	R: 5′CCAGCCCGTCAGATCTCCTTGT′
GAPDH	F: 5′ GGGTCCCAGCTTAGGTTCAT3′
	R: 5′TACGGCCAAATCCGTTCACA3′

### Gene knockdown assays

The Nurr1 small interfering RNA (siRNA) and scramble RNA (scRNA) sequences were designed by using iScore database. The Nurr1 siRNA sense sequence was (5′rCrUrArCrCrUrGrArArArUrUrGrGrArArGrArCrUrUrGrGTA3′) and antisense sequence (5′ rUrArCrCrArArGrUrCrUrUrCrCrArArUrUrUrCrArGrGrUrArGrArA 3′); scRNA sense sequence was (5′ rCrGrUrUrArArUrCrGrCrGrUrArUrArArUrArCrGrCrGrUAT 3′) and antisense sequence (5′rArUrArCrGrCrGrUrArUrUrArUrArCrGrCrGrArUrUrArArCrGrArC 3′). Cells were transfected with 25 nM siRNA and scRNA using Lipofectamine® RNAiMAX (cat. no. L2448202; Invitrogen) after reaching 60–70% confluence for 24h at 37̊C then treated with IAAH-NPs or LPS treatment. Nurr1 expression levels from knockdown were assessed by RT-PCR.

### ELISA and release of secretory pro-inflammatory cytokines

Following the manufacturer’s instructions of Quantikine ELISA Kit (R&D Systems), SimA9 cells were treated as indicated and the media was measured to determine the concentration of secreted cytokines TNF-α and IL-6. Results were arranged, and released cytokines concentrations were calculated using excel, and the results were further analyzed using GraphPad Prism 8 software for statistical analysis.

### Statistical analysis

GraphPad Prism 8 software was used to measure the IC_50_ of tested for pegylated NPs using the logarithmic trend line of cytotoxicity graphs (log (concentration versus inhibition)). Gene expression was presented as mean ± SEM. Differences were compared using the unpaired student’s t-test. A P-value of less than 0.05 will be considered statistically significant.

## Results

### Modeling interaction of IAAH with Nurr1

**Figure 2:**
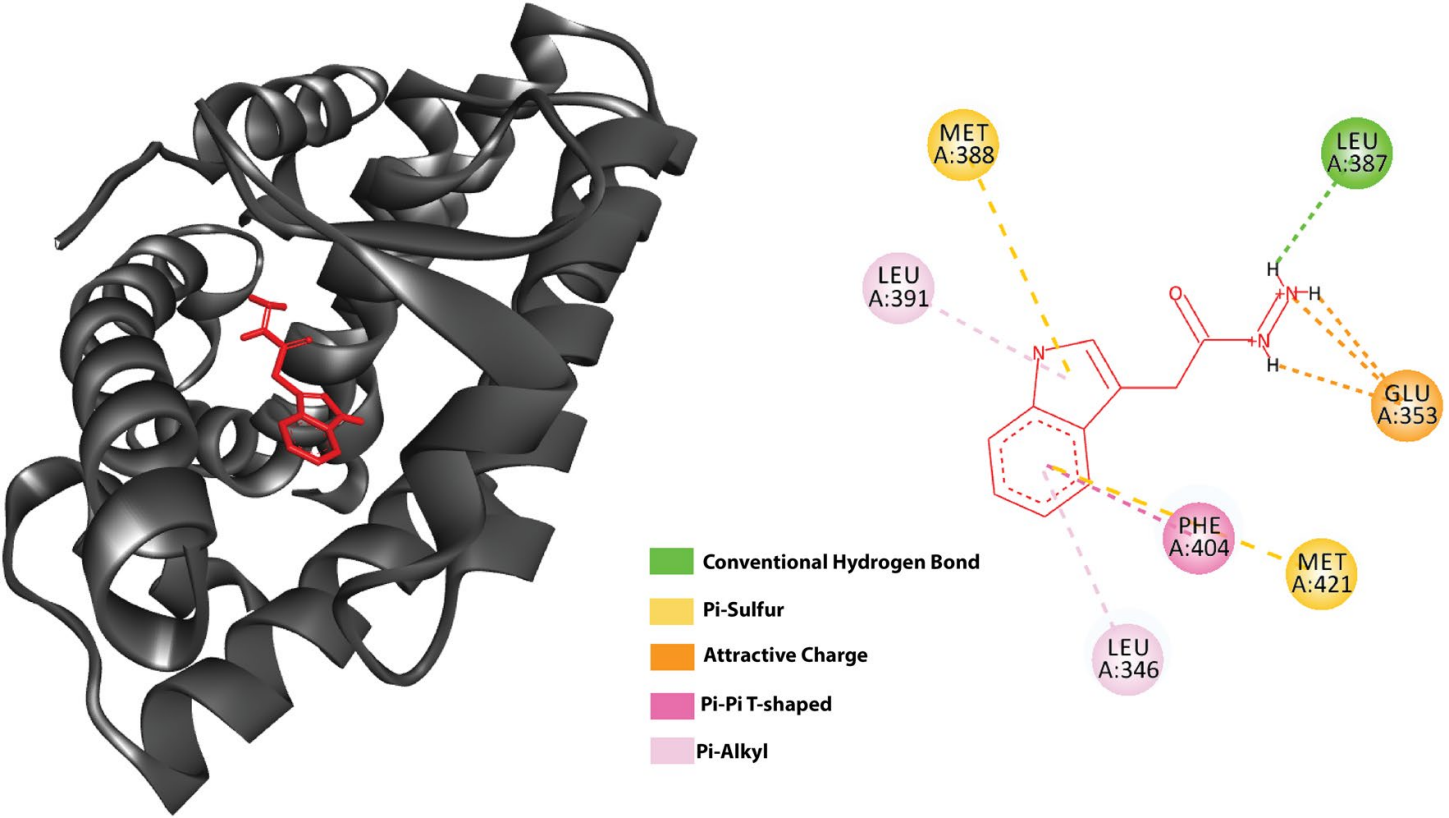
Molecular docking simulations of IAAH with Nurr1. (**A**) IAAH docked ribbon representation of Nurr1 LBD, (**B**) represent binding site residues in Nurr1 that bond to IAAH.

To investigate the possibility of IAAH binding to Nurr1 several molecular docking simulations were performed. The 3D structure of Nurr1 was obtained from the PDB website, a crystal structure of Nurr1 bound to dopamine was chosen as dopamine is similar in structure and size to IAAH. Since the exact mechanism of IAAH binding to Nurr1 is unknown, a blind docking approach was followed, where IAAH was allowed to bind to any position on the protein surface. Due to computational limitations, a rigid protein structure was used, while allowing IAAH to be flexible to rotate when possible. Autodock 4 was used to run the docking simulations. Then ligand models where the IAAH bound to the same ligand binding site reported in the literature were chosen^17^. Consistent with Hammond results, Nurr1 was bound to IAAH with a calculated binding energy of -4.85kcal/mol **(**Fig. 2), with the following residues involved in interaction: THR:595, SER:441, PRO:597, CYS:566, ARG:515, and HIS:516, IAAH has bound almost identically to these same residues.

### Characterization of IAAH-NPs

**Figure 3:**
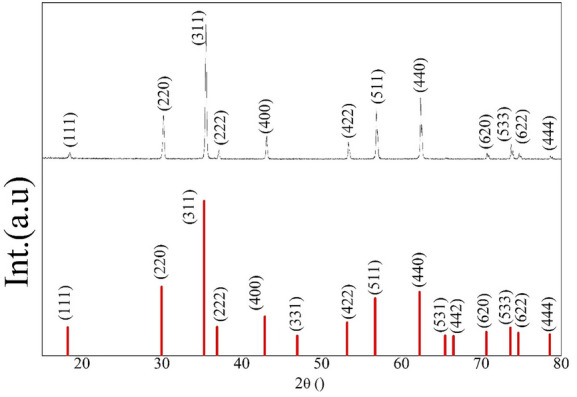
XRD patterns of (**A**) standard ICSD card and (**B**) prepared ZnFe_2_O_4_ NPs.

**Figure 4:**
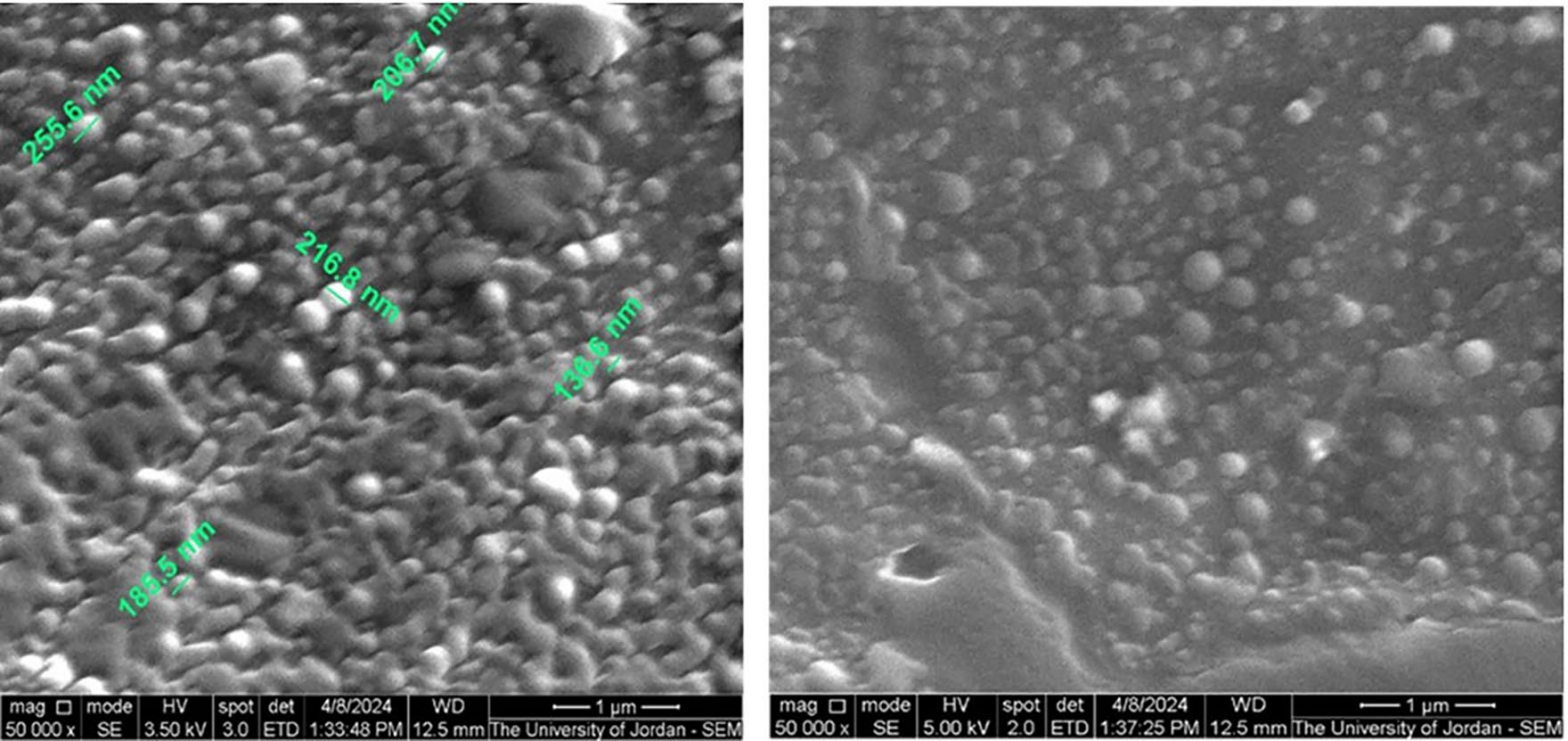
SEM of the IAAH with ZnFe2O4 NPs, IAAH-NPs.

**Figure 5:**
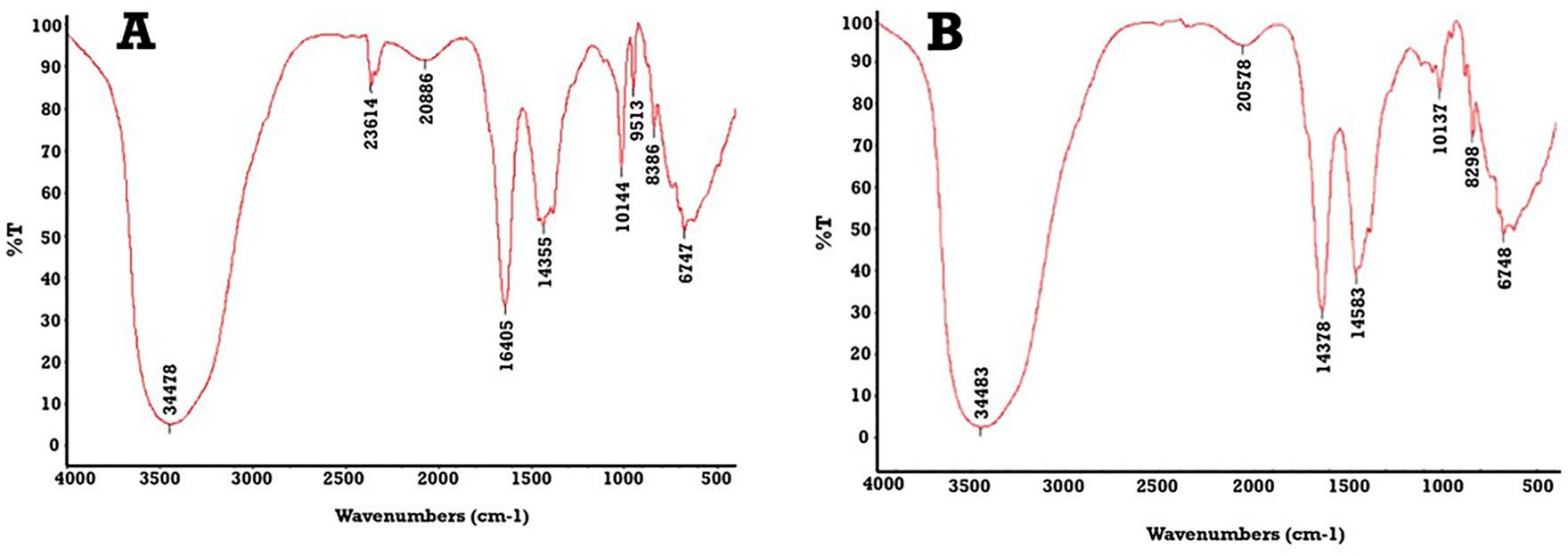
the FTIR of PEGylated nanoparticles. (**a**) IAAH-NP and (**b**) IAAH-ZnFe2O4 NPs.

The phase crystallinity of ZnFe_2_O_4_ NPs was initially determined by the XRD pattern. Figure 3 shows the XRD patterns of ZnFe_2_O_4_ that exhibited intense peaks of spinel ZnFe_2_O_4_ phase that matched well with the standard ICSD card No. 00–001-1108. Results here confirm the crystalline nature of ZnFe_2_O_4_ NPs, where the atoms are arranged in a periodic pattern of three dimensions with an average size of 52.53 nm. The structure and morphology of the synthesized nanoparticles are shown in the SEM image (Fig. 4a**)** and compared to the morphology of the ZnFe_2_O_4_. From these images the structure of the nanoparticles was found to monodispersed nanoparticles were clearly observed from the SEM image shows particle sizes between 100 and 290 nm and the for the ZnFe_2_O_4_ is 50-73 nm (Fig. 4b). Furthermore, the two particles are analyzed via FTIR (Fig. 5**)** and it present a change between the free ZnFe_2_O_4_ and the coated in presence of IAAH.

**Figure 6:**
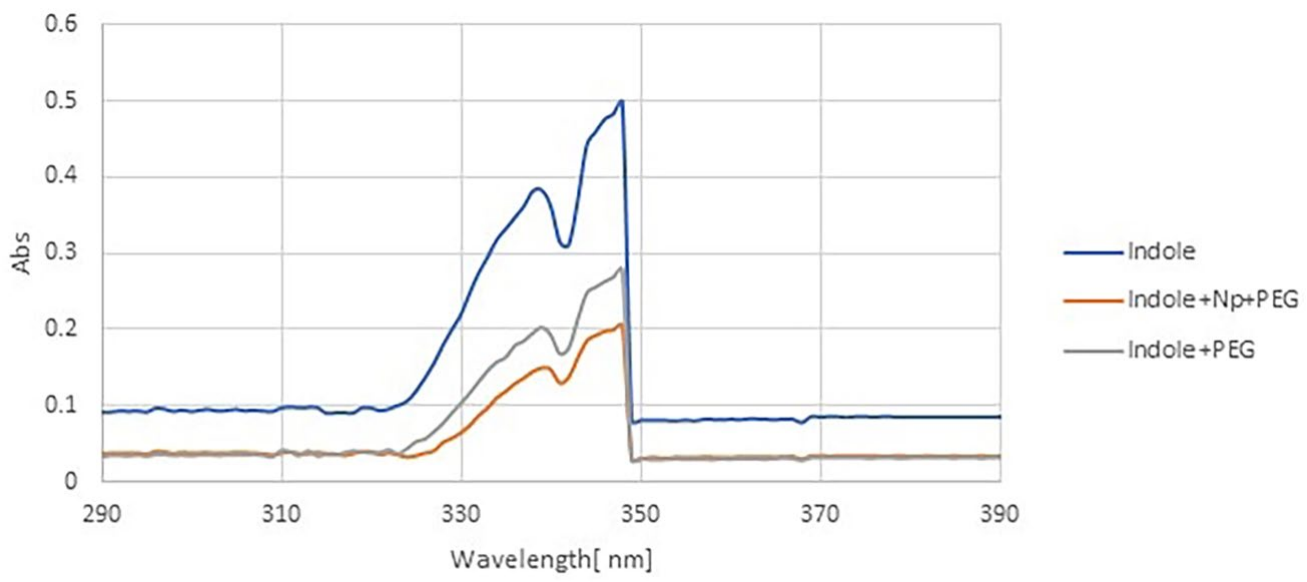
UV–visible spectroscopy of IAAH and IAAH-NPs.

Investigating the conjugation of IAAH with ZnFe_2_O_4_ NPs, the drug-loaded nanoparticles were characterized by UV–visible spectroscopy. Figure 6 shows firm peaks at about 348 nm. The efficiency of IAAH loading into ZnFe_2_O_4_ NPs was calculated from the characteristic absorption bands of initial IAAH and non-conjugated IAAH, as indicated below. It was observed that 72.96% of the IAAH was loaded on ZnFe2O4 NPs. UV–visible spectroscopy was calibrated on the water as blank before measuring the capsulation.

$${\text{Efficiency }} = \, \left( {\left( {{\text{total amount of IAAH }} - {\text{ free IAAH}}} \right) \, /{\text{ total amount of IAAH}}} \right) \, \times { 1}00\%$$

Using zeta potential analysis, we measured IAAH-NPs potential of + 39 mV and an average particle size of 322.67 nm. This high positive charge creates a repulsion between particles with a high degree of stability with low aggregations. The Polydispersity index (PDI) value of 0.19 which range from 0 to 1 reflects a monodisperse phase of IAAH-NPs.

### Effect of IAAH-NPs on viability of SimA9 cells

IAAH-NPs were tested for their safety and applicable dose on SimA9 cells. It was found that the IC_50_ of IAAH-NPs was 50.13 ± 10.53 µg/ml compared with untreated cells and the concentrations below 20µg/ml of IAAH-NPs did not affect the viability of SimA9 cells; therefore, these concentrations were chosen for further investigations.

### IAAH-NPs decrease the pro-inflammatory gene expressions in SimA9 microglia

**Figure 7:**
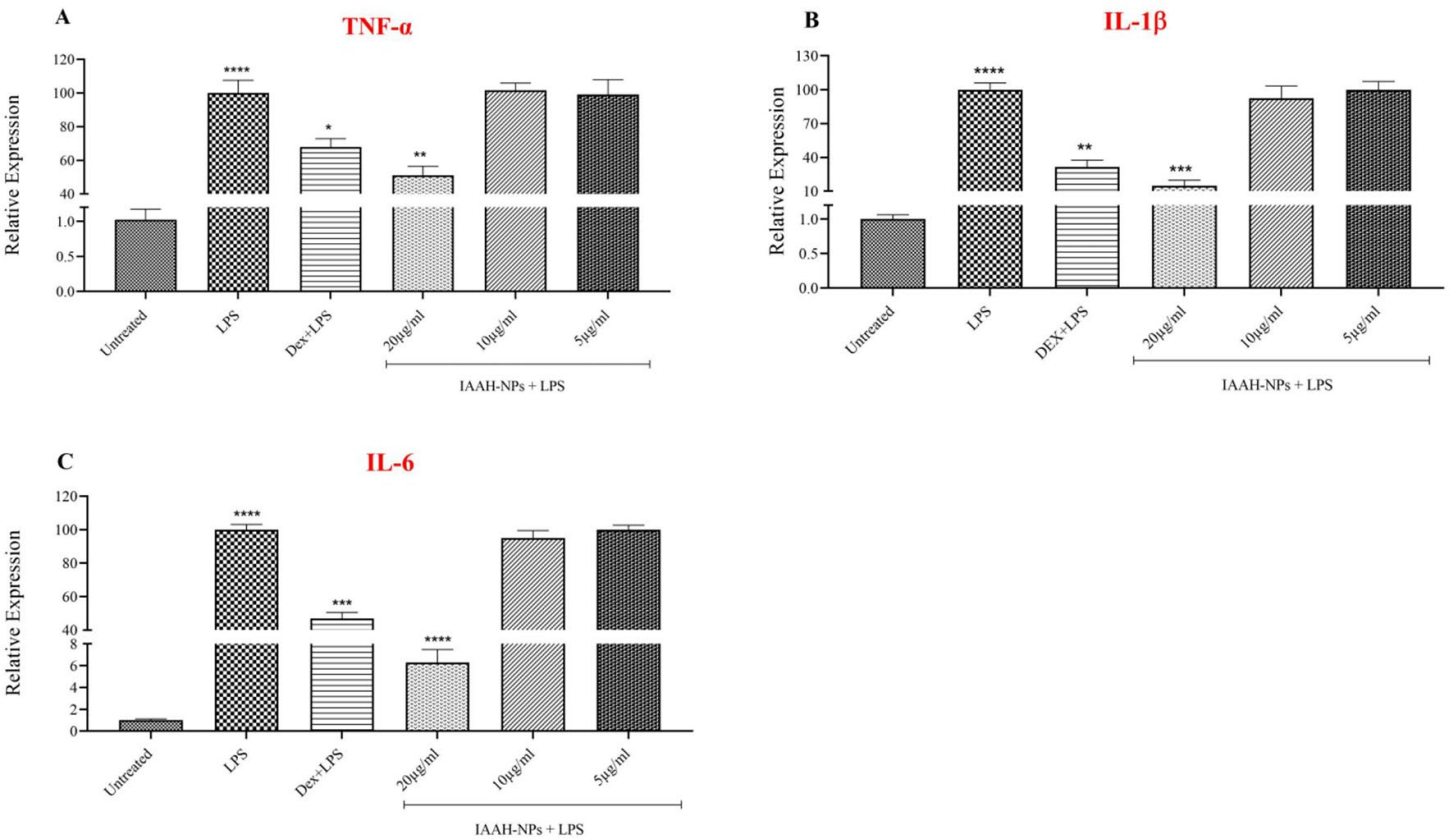
Anti-inflammatory effect of IAAH-NPs on mRNA level of pro-inflammatory cytokines. SimA9 cells were pretreated for 1h with selected concentrations of IAAH-NPs 20, 10, and 5µg/ml followed by 6h of LPS (100ng/ml) then the expression levels of (**A**) TNF-α, (**B**) IL-1β, and (**C**) IL-6 cytokines were measured. Results are presented as the relative expression (%) compared to the LPS-treated group. *p < 0.05, **p < 0.01, ***p < 0.001, ****p < 0.0001.

The mRNA level of pro-inflammatory cytokines including TNF-α, IL-1β, and IL-6, inflammatory markers of LPS-stimulated microglia, were investigated using RT-PCR. We found that the gene expression of pro-inflammatory cytokines significantly increased TNF-α (*p* < 0.0004), IL-6 (*p* < 0.0001), and IL-1β (*p* < 0.0001) in response to LPS at 100ng\ml after 6h exposure compared to untreated cells. In contrast, pre-treatment with IAAH-NPs for 1h inhibited the induction of cytokines expression by LPS stimulation. Results are illustrated in Fig. 7 and demonstrated that IAAH-NPs significantly reduced expression of the three tested cytokines: TNF-α (Fig. 5A), IL-1β (Fig. 5B), and IL-6 (Fig. 5C) at the highest concentration 20µg/ml in comparison to their expression with LPS treatment. The percentage of expression inhibition of TNF-α, IL-1β, and IL-6 was 48.8, 85, and 90.5%, respectively. However, a concentration of 10µg/ml IAAH-NPs failed to change the expression of TNF-α and IL-6 while IL-1β expression levels were reduced with a percentage of 7.7%, but this reduction did not reach statistical significant. For the lowest concentration of IAAH-NPs, no significant anti-inflammatory activity over the tested cytokines was observed.

**Figure 8:**
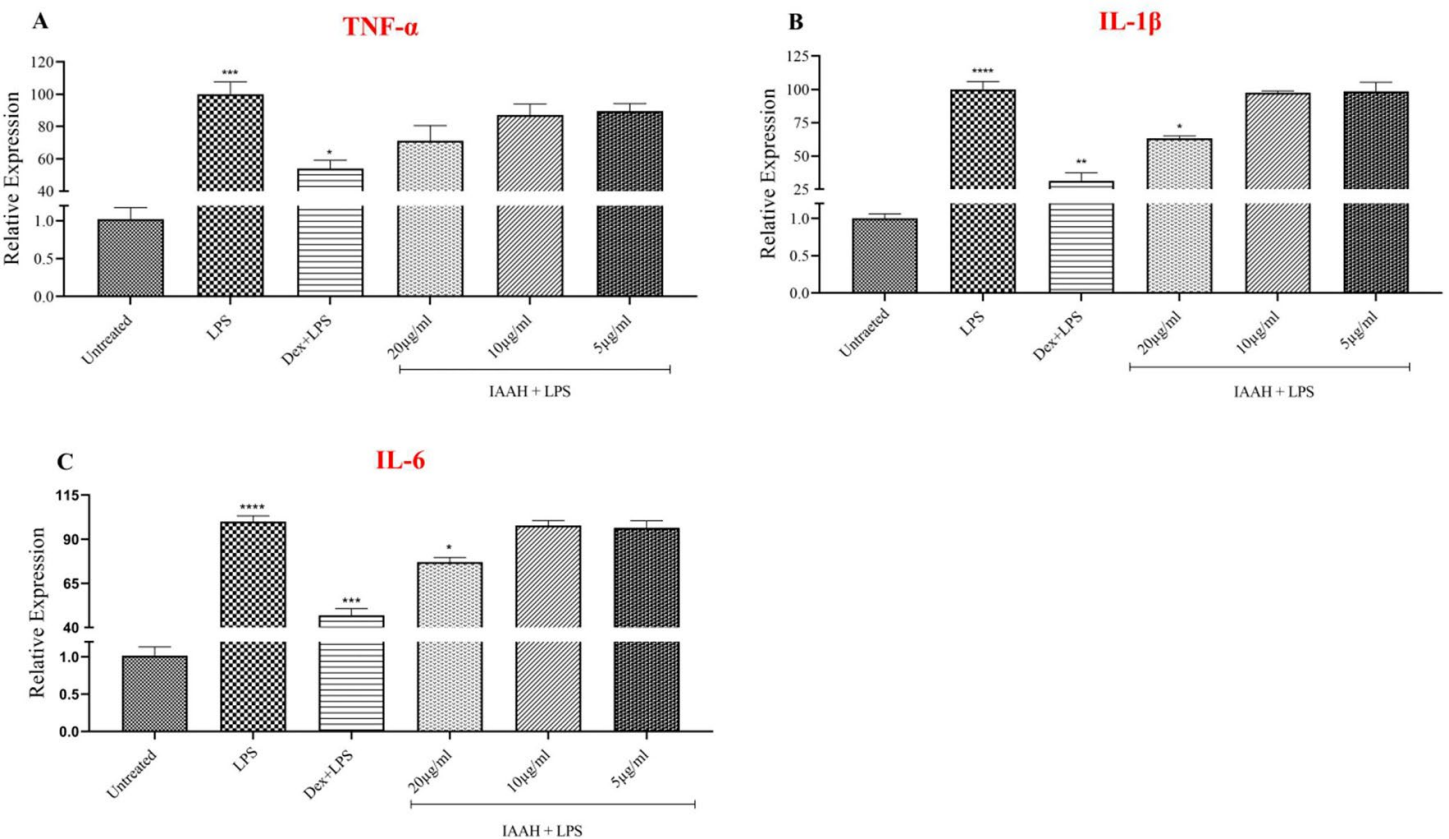
Anti-inflammatory effect of IAAH-NPs on mRNA level of pro-inflammatory cytokines. SimA9 cells were pretreated for 1h with selected concentrations of IAAH 20, 10, and 5µg/ml followed by 6h of LPS (100ng/ml). Dexamethasone was used as a positive anti-inflammatory control with a concentration of 40µg/ml. The level of expression of (**A**) TNF-α, (**B**) IL-1β, and (**C**) IL-6 cytokines was measured. Results are presented as the relative expression (%) of each treatment group in comparison to the LPS-treated group. *p < 0.05, **p < 0.01, ***p < 0.001, ****p < 0.0001.

On the other hand, treated cells with IAAH before LPS demonstrate that TNF-α expression level was not inhibited at any of the three concentrations in comparison to the LPS-stimulated cells **(**Fig. 8A**).** Interestingly, IL-1β expression levels were significantly reduced at a concentration of 20µg/ml with a percentage of 36.8% in comparison to LPS-stimulated cells with (*p* = 0.0180). While at both concentrations 10 and 5µg/ml, there was no significant reduction in IL-1β gene expression levels (Fig. 8B**).** In addition, IL-6 expression levels were significantly reduced at a concentration of 20µg/ml with a percentage of 31.23% in comparison to LPS-stimulated cells with (*p* = 0.0147). In contrast, the other two concentrations showed no significant changes in IL-6 expression levels (Fig. 8C**).** Notably, dexamethasone, a positive anti-inflammatory control, inhibited the expression of the three cytokines TNF-α, IL-6, and IL-1β with a percentage of 32.06, 53.06, and 68.47%, respectively, in comparison to LPS with (*p* = 0.0233), (*p* = 0.0004), and (*p* = 0.0012) respectively.

### IAAH-NPs decrease pro-inflammatory proteins released in SimA9 microglia

**Figure 9:**
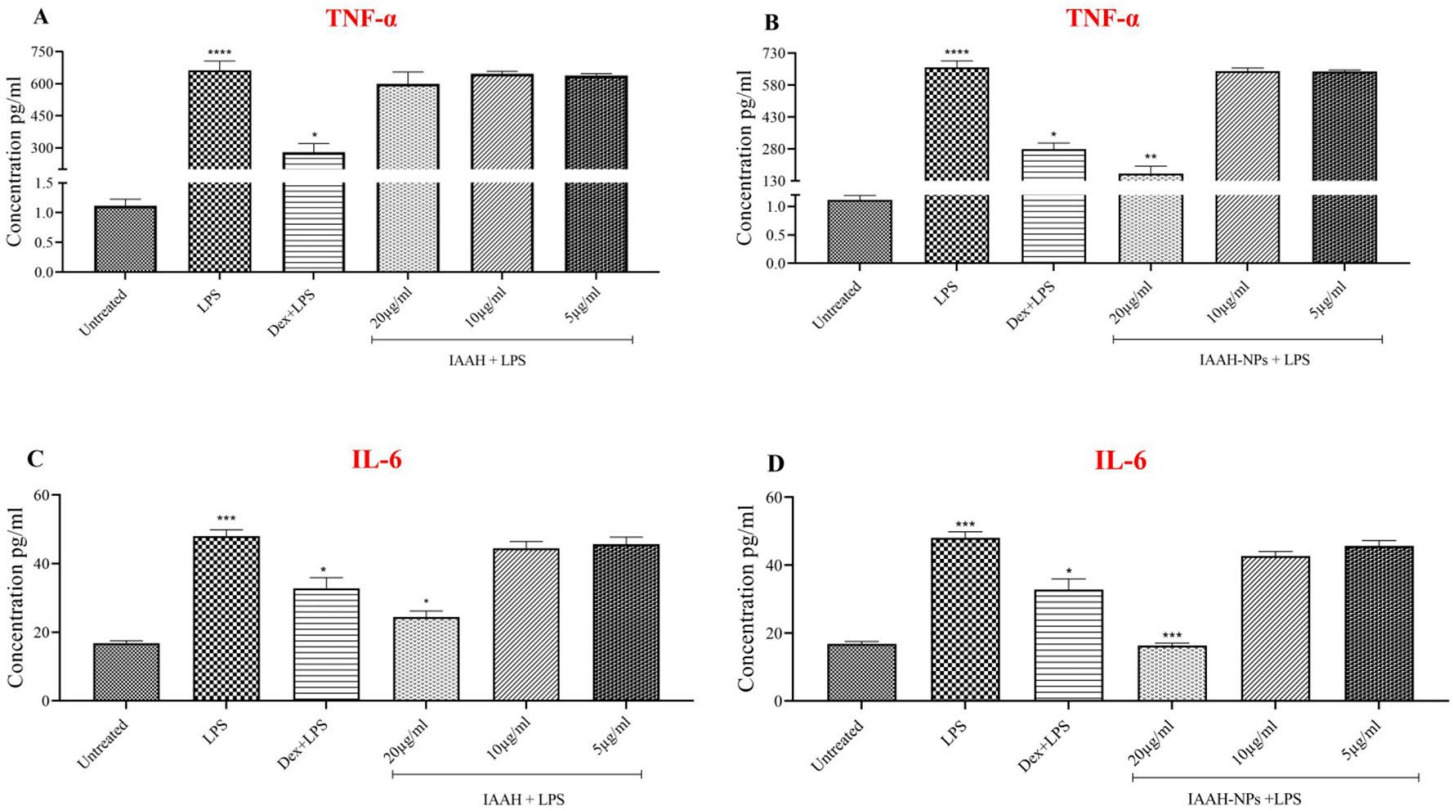
Anti-inflammatory effect of IAAH and IAAH-NPs on the release of TNF-α and IL-6 pro-inflammatory cytokine. SimA9 cells were treated with three different concentrations of IAAH and IAAH-NPs for 1h prior and then activated with LPS for 6h then ELISA was used to measure the concentration of TNF-α release (A and B) and IL-6 (C and D). Results are presented as concentration (pg/ml) and compared to the LPS-treated group. *p < 0.05, **p < 0.01, ***p < 0.001, ****p < 0.0001.

Results show that IAAH-NPs at a concentration 20µg/ml significantly dropped the TNF-α level from 662.32pg/ml in the LPS-treated group to 164.58pg/ml in the IAAH-NPs treated group with (*p* = 0.0086). In contrast, the naked IAAH failed to reduce the level of TNF-α (Fig. 9A, B**)**. This data confirms the IAAH-NP effect on mRNA levels and highlights their anti-inflammatory effect activity over tested microglia. In comparison to IAAH, ZnFe_2_O_4_ NPs as delivery vehicles increase the IAAH efficiency by increasing its concentration inside the cell.

For IL-6 release, IAAH-NPs at a concentration 20µg/ml significantly dropped the IL-6 levels from 48pg/ml for the LPS-treated group to 16.28pg/ml for the IAAH- NPs treated group with (*p* = 0.0003). In contrast, the naked IAAH failed to reduce the level of IL-6 significantly (Fig. 9C, D**)**. This data confirms the IAAH-NPs effect on mRNA levels and emphasizes their anti-inflammatory effect activity over tested microglia.

### IAAH-NPs up-regulated Nurr1 expression on LPS-stimulated SimA9

**Figure 10:**
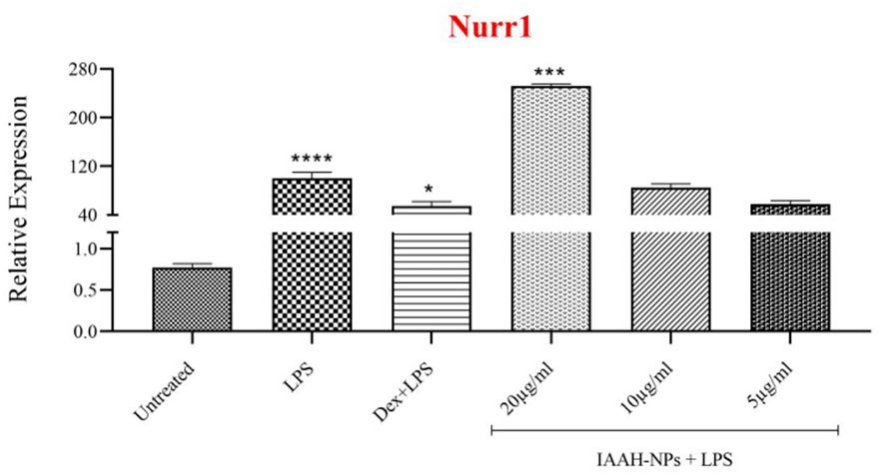
Up regulation of Nurr1 mRNA level after treatment with IAAH-NPs. SimA9 cells were pretreated for 1h with selected concentrations of IAAH-NPs followed by 6h of LPS (100ng/ml) then the expression level of Nurr1 was measured. Results are presented as the relative expression (%) compared to the LPS-treated group. *p < 0.05, **p < 0.01, ***p < 0.001, ****p < 0.0001.

To determine the Nurr1 involvement as a modulator of the inflammation mechanism, the expression levels of Nurr1 were measured. The data showed a reduction in Nurr1 expression in LPS-stimulated cells compared to untreated cells with (*p* = 0.0856). Interestingly, IAAH-NPs elevated the expression of Nurr1 significantly at a concentration of 20µg/ml with (*p* = 0.0001) compared to the LPS-treated group and with (*p* = 0.0110) in comparison to untreated cells. However, at both concentrations 10 and 5µg/ml failed to increase Nurr1 expression levels compared to LPS-treated cells Fig. 10.

### IAAH-NPs require Nurr1 expression to suppress pro-inflammatory cytokines released on LPS-stimulated SimA9

**Figure 11:**
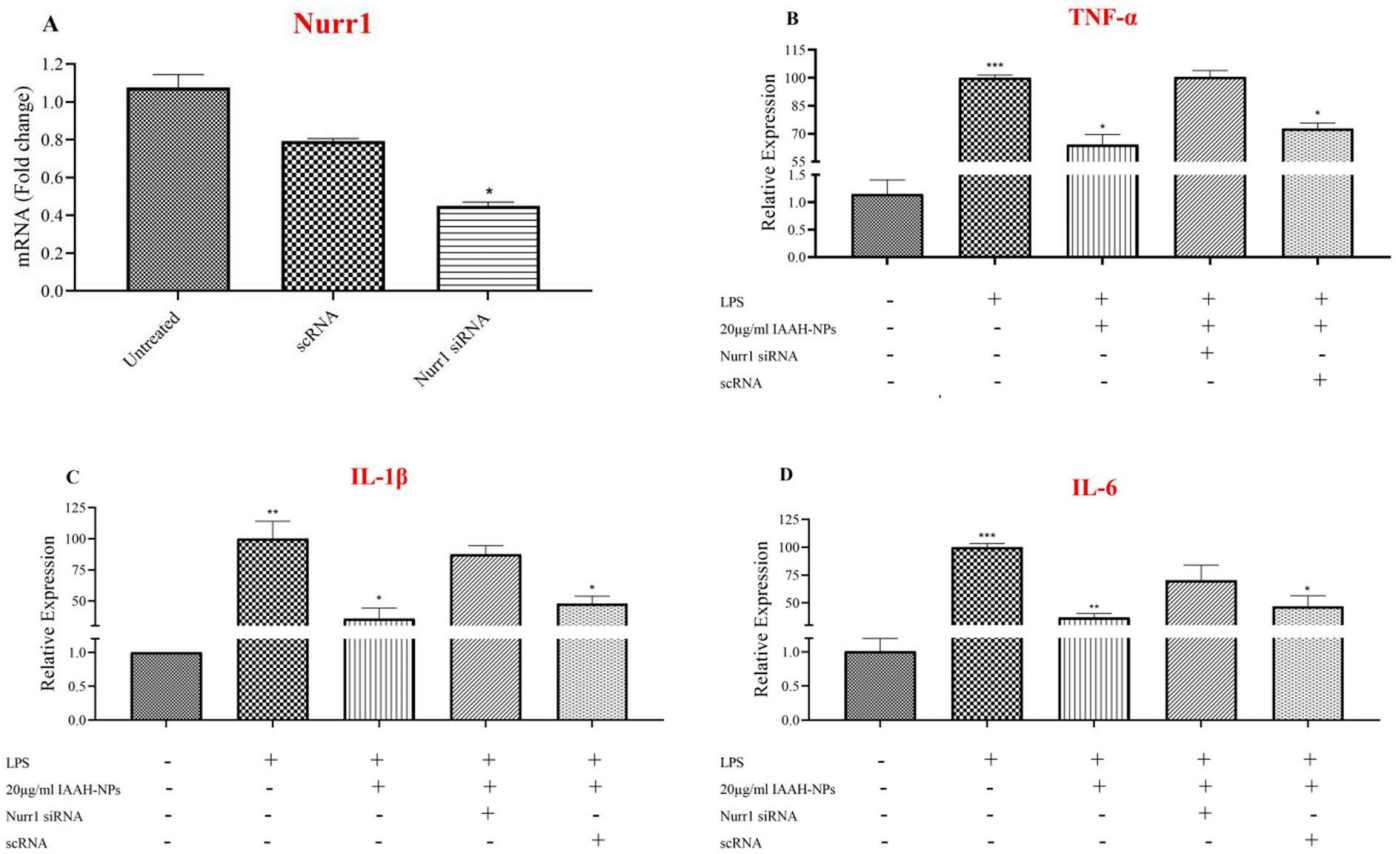
IAAH-NPs dependent inhibition of pro-inflammatory gene expressions requires Nurr1 expression. SimA9 cells were treated with sequence of siRNA results in decrease of Nurr1 expression levels. Results are presented as the relative expression (%) compared to the LPS-treated group. *p < 0.05, **p < 0.01, ***p < 0.001, ****p < 0.0001.

To study Nurr1 involvement in pro-inflammatory cytokines suppression by IAAH-NPs, Nurr1 knockdown was applied by siRNA. The expression level of Nurr1 was decreased significantly with (*p* = 0.0217) compared to untreated cells after 25nM siRNA treatment **(**Fig. 11A**)**. The pro-inflammatory gene expression levels were measured after Nurr1 knockdown and IAAH-NPs or LPS treatments. The results indicated that the LPS-stimulated SimA9 cells that were treated with IAAH-NPs failed to suppress the pro-inflammatory cytokines gene expression levels including TNF-α, IL-1β, and IL-6 **(**Fig. 11B,C,D**)**. These data show that IAAH requires the expression of Nurr1.

## Discussion

Designing a delivery system for treatment has been developed rapidly. In the current work, ZnFe_2_O_4_ NPs were synthesized first and then IAAH was loaded into these NPs then coated with PEG. Keep in mind that the coating material conferred excellent thermal stability and recyclability, so for this reason PEG was employed as a coating material for all tested samples^18^. The results revealed the formation of crystals for IAAH-NPs with an average size of 322.6 nm, while the average size of IAAH-ZnFe_2_O_4_ NPs was measured at 210 nm. Subsequently, Fourier-transform infrared spectroscopy (FTIR) analysis was conducted on the coated NPs of IAAH and IAAH-ZnFe2O4 and in both it confirm the coating observed around 1100–1200 cm^−1^, corresponding to the C–O–C stretching vibration of the ether linkage in PEG molecules. Additionally, peaks in the region of 2800–3000 cm^−1^ indicate the presence of C–H stretching vibrations, characteristic of both the PEG coating and the nanoparticle core. the presented zeta potential data showed a high positive charge of IAAH-NPs that confirm the polymeric stability where the repulsion process prevents the polymeric aggregation besides increasing the cellular attraction. Hence, this positive charge is thought to be a factor in increasing the acidity after endocytosis, where this will help to escape the lysosomal factor^18^.

Since NPs work as a delivery system, ZnFe_2_O_4_ NPs with a positive charge create an interaction with the cell membrane that helps to enter the cell; consequently, increasing the efficiency of IAAH by raising the intracellular uptake. Another importance of NPs is the ability to escape the lysosome after endocytosis, where ZnFe_2_O_4_ NPs create an acid environment inside the lysosome that creates pores that allow IAAH leakage and escape the elimination^18^. These results suggest the high intracellular uptake of IAAH-NPs increased the cell’s IAAH concentration.

Microglia cells are noteworthy to be investigated due to their role in neuroinflammation mechanism. Therefore, Nurr1 has been recognized as a trans-repressor for pro-inflammatory mediators where its overexpression suppresses inflammation while the knockdown of Nurr1 enhances inflammation^2,19^. Several ligands were identified as Nurr1 agonists; however, their anti-inflammatory effects have not been tested yet. Based on many previous studies which deal with Nurr1 ligands and their structures, we synthesized a new formula of a chemical compound, IAAH, and proposed that IAAH is an activator of Nurr1 receptor. Furthermore, to enhance the efficiency of this newly proposed ligand, a special delivery system was designed for this goal, and the effect of this compound in manipulating neuro-inflammatory responses by acting on Nurr1 was investigated on SimA9 microglia cells^7^. Findings manifest the ability of IAAH-NPs to mitigate inflammation by testing the changes in pro-inflammatory cytokines gene expression levels including TNF-α, IL-1β, and IL-6.

In a previous study, AQ and CQ were examined for their anti-inflammatory effects in LPS-stimulated BV2 microglia that showed a reduction of TNF-α, IL-6, and IL-1β, but lack of specificity to this receptor was a hurdle^6,20^. In another study, C-DIM12 reduced the production of TNF-α and IL-6 significantly while IL-1β might need a higher concentration to obtain a significant reduction^7^. In the current study, the presented results from measuring the gene expression levels of pro-inflammatory cytokines demonstrate that IAAH at a concentration of 20µg/ml significantly reduces the expression of IL-1β and IL-6 which is consistent with De Miranda results except for TNF-α cytokine, which did not show any notable reduction. In comparison to naked IAAH, IAAH loaded into ZnFe_2_O_4_ NPs showed spectacular efficiency in delivering IAAH and inhibiting TNF-α, IL-1β*,* and IL-6 production. It is worth mentioning that the efficiency of IAAH was achieved by its coating with ZnFe_2_O_4_ NPs and PEG as a high efficient delivery system, which remarkably enhanced the cellular uptake of IAAH, evidenced by the profound anti-inflammatory effect of NPs and PEG coated IAAH (Fig. 7) compared to the reduced anti-inflammatory effect of naked IAAH (Fig. 8). Moreover, to confirm the anti-inflammatory activity of IAAH-NPs on the release of pro-inflammatory cytokines, ELISA was performed on TNF-α and IL-6. Results demonstrate the inhibition of TNF-α and IL-6 at 20µg/ml concentration of IAAH-NPs which is consistent with the gene expression results.

Based on previous findings, the Nurr1 association in neuroinflammation inhibition was evaluated by measuring its expression as a positive feedback loop in response to inflammation^21,22^. Our direct evidence for the involvement of Nurr1 in neuroinflammation is its significant increase in microglial cells upon their exposure to LPS. However, the expression of Nurr1 was further elevated when microglial cells were pretreated with IAAH-NPs before being stimulated with LPS. These data suggest that IAAH-NPs play an anti-inflammatory role not only through its binding to Nurr1 to activate it, but also through its positive effect on the expression of Nurr1 itself in a positive feedback loop of binding and enhancement of Nurr1 expression, which in turn play a role in the inhibition of pro-inflammatory cytokines. It has been postulated before that Prostaglandin E2 (PGE2) is an agonist for Nurr1, which interact to and enhance activity of Nurr1[23]. Interestingly, PGE2 was also found to enhance the expression of Nurr1 through a sequential activation events that converge to activate CREB, which in turn binds to its response element which is located in Nurr1 promoter, representing a positive feedback loop of sequential activation of both activity and expression of Nurr1 [24]. Accordingly, we suggest that our compound follows the same pattern of sequential events leading to a positive feedback loop of activation and expression. Our data suggest the involvement of IAAH-NPs in pro-inflammatory cytokines inhibition. In contrast, De Miranda’s result showed that C-DIM12 decreases Nurr1 expression which might refer to the differences of the type of cells, as they used BV2 cells, in addition to the longer period of LPS exposure, which is 24h in De Miranda’s experiment compared to 6 h only in ours. This suggest that our IAAH-NPs are more potent and fast acting ligand of Nurr1. However, a time course study is needed to determine the Nurr1 expression upon IAAH-NPs and LPS treatment^22^. To confirm that IAAH-NPs exert their anti-neuroinflammatory effect through binding and activating Nurr1, mRNA of Nurr1 was knocked-down using siRNA technology. Our data indicate that Nurr1 knock-down lead to an elevation of pro-inflammatory cytokines expression even in the presence of IAAH-NPs. These results were consistent with previous reports that support the role of Nurr1 as an anti-inflammatory mediator by blocking the inflammatory gene expressions^2,7^.

Another key point, the molecular docking simulation results initially support our claim that IAAH-NPs ability to inhibit the pro-inflammatory cytokines is due to IAAH binding to the Nurr1 receptor. It is worth mentioning that in the in silico analysis, IAAH was able to dock outside the ligand binding site as well, but whether these interactions are meaningful or not, further analysis must be carried out in the future. The binding to the ligand binding site could be confirmed by using an assay with a competitive inhibitor/activator. In case the ligands were not binding at the ligand binding site, other allosteric effects must be assayed to reveal the exact molecular mechanism.

In conclusion, nanotechnology opens the door to improving drug delivery. The present work highlights the importance of nanoparticles to increase drug efficiency, where this technology is worthy of delivering IAAH. In IAAH-NPs pretreated microglia, the LPS-induced TNF-α, IL-1β, and IL-6 production was impressively suppressed and notably elevated Nurr1 expression levels giving an insight into Nurr1 involvement. Inhibition of Nurr1 expression indicated its requirement to suppress pro-inflammatory mediators’ output. Taken together, these results suggest that IAAH-NPs combining IAAH with ZnFe_2_O_4_ as a delivery system might have a therapeutic potential in neuroinflammation-related neurodegenerative disorders through the down regulation of microglia-mediated inflammatory response via the Nurr1 receptor.

## Data Availability

All data generated or analysed during this study are included in this published article.
