# Author Correction: The Nurr1 ligand indole acetic acid hydrazide loaded onto ZnFe2O4 nanoparticles suppresses proinflammatory gene expressions in SimA9 microglial cells

**DOI:** 10.1038/s41598-024-69679-8

**Published:** 2024-08-15

**Authors:** Raneen Qasim, Tuqa Abu Thiab, Tareq Alhindi, Afnan Al‑Hunaiti, Amer Imraish

**Affiliations:** 1 Department of Biological Sciences, School of Science, The University of Jordan, Queen Rania Al‑Abdullah Street, Amman, 11942 Jordan; 2 Department of Chemistry, School of Science, The University of Jordan, Queen Rania Al‑Abdullah Street, Amman, 11942 Jordan

Correction to: *Scientific Reports* 10.1038/s41598-024-64820-z, published online 17 June 2024

In the original version of this Article, Afnan Al-Hunaiti was incorrectly listed as a corresponding author. The correct corresponding author for this Article is Amer Imraish. Correspondence and request for materials should be addressed to a.imraish@ju.edu.jo.

The original Article has been corrected.

